# Three-dimensional balanced steady state free precession myocardial perfusion cardiovascular magnetic resonance at 3T using dual-source parallel RF transmission: initial experience

**DOI:** 10.1186/s12968-014-0090-0

**Published:** 2014-11-28

**Authors:** Roy Jogiya, Andreas Schuster, Arshad Zaman, Manish Motwani, Marc Kouwenhoven, Eike Nagel, Sebastian Kozerke, Sven Plein

**Affiliations:** King’s College London BHF Centre of Excellence, NIHR Biomedical Research Centre and Welcome Trust and EPSRC Medical Engineering Centre at Guy’s and St. Thomas’ NHS Foundation Trust, Division of Imaging Sciences, The Rayne Institute, London, SE1 7EH UK; Multidisciplinary Cardiovascular Research Centre & Leeds Institute of Cardiovascular and Metabolic Medicine, University of Leeds, Leeds, LS2 9JT UK; Philips Healthcare, Best, Eindhoven, The Netherlands; Institute for Biomedical Engineering, University and ETH Zurich, Zurich, Switzerland; Department of Cardiology and Pneumology and German Centre for Cardiovascular Research (DZHK, Partner Site Göttingen), Georg-August-University, Göttingen, Germany

**Keywords:** Myocardial perfusion, Cardiovascular magnetic resonance, Whole heart imaging, Balanced steady state free precession, Artefact

## Abstract

**Background:**

The purpose of this study was to establish the feasibility of three-dimensional (3D) balanced steady-state-free-precession (bSSFP) myocardial perfusion cardiovascular magnetic resonance (CMR) at 3T using local RF shimming with dual-source RF transmission, and to compare it with spoiled gradient echo (TGRE) acquisition.

**Methods:**

Dynamic contrast-enhanced 3D bSSFP perfusion imaging was performed on a 3T MRI scanner equipped with dual-source RF transmission technology. Images were reconstructed using *k*-space and time broad-use linear acquisition speed-up technique (*k-t* BLAST) and compartment based principle component analysis (*k-t* PCA).

**Results:**

In phantoms and volunteers, local RF shimming with dual source RF transmission significantly improved B1 field homogeneity compared with single source transmission (P = 0.01). 3D bSSFP showed improved signal-to-noise, contrast-to-noise and signal homogeneity compared with 3D TGRE (29.8 vs 26.9, P = 0.045; 23.2 vs 21.6, P = 0.049; 14.9% vs 12.4%, p = 0.002, respectively). Image quality was similar between bSSFP and TGRE but there were more dark rim artefacts with bSSFP. *k-t* PCA reconstruction reduced artefacts for both sequences compared with *k-t* BLAST. In a subset of five patients, both methods correctly identified those with coronary artery disease.

**Conclusion:**

Three-dimensional bSSFP myocardial perfusion CMR using local RF shimming with dual source parallel RF transmission at 3T is feasible and improves signal characteristics compared with TGRE. Image artefact remains an important limitation of bSSFP imaging at 3T but can be reduced with *k-t* PCA.

**Electronic supplementary material:**

The online version of this article (doi:10.1186/s12968-014-0090-0) contains supplementary material, which is available to authorized users.

## Background

Two-dimensional (2D) myocardial perfusion cardiovascular magnetic resonance (CMR) is increasingly used as a clinical tool for the assessment of myocardial ischemia [1,2]. In recent years, 3T MRI scanners have become more widely available and the higher signal to noise ratio (SNR) at this field strength compared with 1.5T is particularly beneficial for myocardial perfusion CMR [3]. In parallel, more sophisticated data acceleration schemes have been introduced, such as *k*-space and time broad-use linear speed up technique (*k-t* BLAST) and *k-*space and time principal component analysis (*k-t* PCA), which in principle allow acceleration of image acquisition ≥10 fold [4]. Together, these developments have facilitated the development of three-dimensional (3D) perfusion CMR [5].

Initial 3D myocardial perfusion studies have used spoiled turbo gradient echo (TGRE) acquisition. Balanced steady state free precession (bSSFP) has become popular for 2D myocardial perfusion CMR at 1.5T because it provides a gain in signal to noise ratio (SNR) of up to 85% and in contrast to noise ratio of up to 50% compared with TGRE [6]. However, bSSFP is more susceptible to artefacts relating to B0 inhomogeneities. In addition, limits on specific absorption rates (SAR) on CMR systems are often addressed by lower B1 amplitudes and hence longer TR, which compromise optimal bSSFP pulse sequences and higher flip angles. All of these effects are more prominent at higher field strength.

A potential solution to these challenges is local RF shimming using dual-source parallel RF transmission, which has been shown to improve image homogeneity, image contrast and diagnostic confidence compared with conventional RF transmission in cardiac cine bSSFP imaging at 3T [7]. The method reduces local SAR hot spots and optimizes the homogeneity of RF deposition. These developments hold particular promise for bSSFP 3D perfusion CMR at 3T.

## Aim

It was the aim of this study to develop an accelerated bSSFP pulse sequence for 3D whole heart myocardial perfusion CMR at 3T using dual-source parallel RF transmission with local RF shimming and to compare it with a TGRE method.

## Methods

### Subjects

The study was approved by the local research ethics committee and all subjects gave written informed consent to participate. Twenty-five healthy volunteers (mean age 28, range 20–57) and five patients (mean age 65, range 49–79) were included (Table 1). Subjects with contraindications to CMR and adenosine were excluded. Patients were recruited prior to clinically indicated invasive coronary angiography studies.

**Table 1:** **Study population demographics**

	**Volunteers**	**Patients**
N	25	5
Male	15 (60%)	4 (80%)
Age, Years	28.0 +/− 8.1	64.6 +/−13.7
Range	20-57	49-79
BMI, kg/m^2^	23.8 +/− 2.6	23.7 +/−1.9

### CMR set up

Subjects were scanned in supine position on a 3T MRI scanner (Achieva 3T-TX, Philips Healthcare, Best, Netherlands) equipped with a Quasar Dual gradient system (60 mT/m; 200 mT/m/ms) and dual-source parallel RF transmission (MultiTransmit) technology [7]. A 6-channel cardiac phased array receiver coil, 4-lead vectorcardiogram, respiratory belt and blood pressure monitoring were used. For each perfusion scan, an intravenous bolus of 0.075 mmol/kg gadobutrol (Gadovist, Bayer, Germany) was administered followed by a 20 ml saline flush (Spectris Solaris power injector, Pittsburgh, Pennsylvania, USA).

### RF Shimming and B1 maps

A cardiac-triggered B1 calibration scan was acquired as one transverse section through the middle of the left ventricle during end-diastole using a saturated double-angle method with a segmented echo-planar imaging readout in a single breath hold. This scan yields the B1 maps for each independent transmit channel (sequentially acquired in the same breathhold), and is used to obtain the phase difference and power ratio of the RF transmit channels for subsequent images with local RF shimming [7].

The RF shim for each scan was determined automatically by vendor-provided MRI scanner software. This software uses an algorithm to minimize the coefficient of signal variation of the B1 field based on the linear combination of phase and amplitude of the two independent RF transmit channels, based on the B1 data from the calibration scan within the user-defined local shim area. An additional B1 map with RF shimming was acquired using the optimized RF shim settings [8].

### Pulse sequence design

In phantom experiments and in five volunteers, a bSSFP pulse sequence was developed to match a previously validated and described TGRE method [5] with the following pulse sequence parameters (TR/TE/flip angle 1.8 ms/0.7 ms/15° with a B1-insensitive tailored composite water suppression enhanced through T1 effects (WET) prepulse, saturation prepulse delay 150 milliseconds, acquisition timed to end systole. Using partial echo and a 75% partial Fourier sampling in the ky-kz direction and an elliptical k-space shutter, 10-fold undersampled *k-t* acquisition with 49 training profiles leading to a net acceleration of 7.0, *k-t* BLAST and *k-t* PCA reconstruction [5], reconstruction of 12 contiguous slices of 5 mm thickness, field of view 350 × 245 mm^2^, acquired voxel size 2.3 × 2.3 × 5 mm^3^, interpolated to 1.5 × 1.5 × 5 mm^3^). Total number of acquired profiles was 106, which results in an acquisition time per heart-beat of 191 ms [106 × 1.8 ms]. Thirty dynamics were acquired in a single inspiratory breath-hold, with every dynamic covering one heart-beat.

Based on these parameters, the bSSFP pulse sequence was optimised to maximise the acquisition flip angle while keeping the TR minimal and the acquisition shot duration to approximately 200 ms. The effect of local RF shimming using dual-source transmission was assessed by quantitative analysis of the B1 field homogeneity. B1 maps were acquired with and without RF shimming. These B1 maps are scaled as a percentage of nominal amplitude. A pixel value of 100 (%) means that the local flip angle corresponds exactly to the nominal (user defined) value.

### Comparison between TGRE and bSSFP acquisition

In the next 15 volunteers, two resting perfusion scans were acquired in random order, using the previously described TGRE [5] and the bSSFP method separated by 20 minutes to allow contrast washout. Table 2 lists the key pulse sequence parameters of the two methods. Imaging was performed in the same plane for both acquisitions, using identical planning. In the last five volunteers of this group, noise maps were generated immediately after each of the contrast enhanced perfusion acquisitions. For this purpose, data were acquired with the excitation flip angle set to “zero” and with reconstruction coefficients inherited from the signal containing acquisition. Signal to noise ratio (SNR) was determined as the ratio of average absolute signal to standard deviation of the real channel of noise in the myocardial region-of-interest of the reconstructed *k-t* PCA images. Contrast-to-noise ratio (CNR) was calculated from the difference between the peak and baseline SNR.

**Table 2:** **Pulse sequence parameters**

	**TGRE**	**bSSFP**
TR	1.8 ms	2.2 ms
TE	0.70 ms	1.04 ms
Flip angle	15°	35°
RF Shimming	Yes (local)	Yes (local)
Partial Echo, Halfscan (Partial Fourier) in x-y and z planes	Yes 0.75/0.75	Yes 0.75/0.75
Spatial resolution	2.3 × 2.3 × 10 mm^3^	2.3 × 2.3 × 10 mm^3^
No slices	12	12
Acquisition	191 ms	211 ms
SAR	<30%	<88%

Homogeneity of the reconstructed perfusion images was calculated in these five subjects based upon a previously described method [9]. Epicardial and endocardial borders (excluding papillary muscles, trabeculation and dark rim artefacts) were drawn in all ventricular slices. Myocardial mean and standard deviation of signal intensity were measured in the dynamic image with peak signal intensity. The coefficient of variation of the myocardial pixel values was measured using the formula: coefficient of variation = standard deviation/mean signal (expressed as a percentage).

### Stress perfusion and detection of coronary artery disease (CAD)

In the last 5 volunteers, bSSFP stress perfusion images were acquired during intravenous adenosine-induced hyperaemia administered for 4 minutes at 140 mcg/kg/min and, following a 20 minute delay, repeated at rest. In addition, in five patients, both TGRE and bSSFP acquisitions at stress were performed in random order. Significant CAD was defined using quantitative coronary angiography (QCA) as >70% diameter stenosis of the left anterior descending, circumflex and right coronary arteries with >2 mm diameter or >50% diameter stenosis for the left main stem as described previously [5].

### Evaluation of image quality and artefacts of MR images

Two experienced observers blinded to clinical details reviewed the CMR images (Extended Workspace, Philips Healthcare, Best, The Netherlands) in a randomised order. The whole heart images were displayed as a complete stack from base to apex and each slice was analysed to produce an overall unified score. Image quality was graded on a scale from 1 to 4 (1 = uninterpretable, 2 = poor, 3 = good, 4 = excellent) for both the standard *k-t* BLAST and reconstructed *k-t* PCA images. Image artefacts were categorised as breathing related, subendocardial dark rim artefacts [10] or related to the reconstruction of undersampled data.

### Statistical analysis

Data were analysed using IBM SPSS, Version 19.0 (SPSS < Chicago, IL, USA). For all analyses P < 0.05 was considered significant. Continuous data were expressed as the mean ± standard deviation. Comparisons between groups were made using two-tailed paired t tests. No corrections were made for multiple comparisons. Image quality scores were compared using the Wilcoxon signed ranks test and McNemar’s test. Discrete data were expressed as percentages.

## Results

Baseline demographics of the 25 volunteers and five patients are given in Table 1. CMR examinations were completed in all subjects and no adverse effects occurred during stress acquisitions.

### Pulse sequence design

Table 3 shows the minimum achievable TR, TE and maximal heart rate at increasing flip angles using the bSSFP sequence in the first five volunteers. The achievable TR shortening and hence the reduction in acquisition time using local RF shimming was very similar between subjects. A flip angle of 35 degrees was found to provide a balanced TR/TE design, a maximal achievable heart rate of 83/min and a shot duration of 211 ms. These settings were used for all subsequent experiments. When switching to single source RF transmission, the same flip angle of 35 degrees required a minimum TR of 3.4 ms (range 3.4-3.5) resulting in a minimum shot duration of 301 ms and leading to increased motion artefact.

**Table 3:** **Pulse sequence optimisation with dual source RF transmission in first five volunteers**

**Flip angle (°/range)**	**Maximal heart rate (bpm)**	**Repetition time (ms)**	**Echo time (ms)**
10 (10–12)	144	1.77	0.76
15 (15–17)	144	1.77	0.76
20 (20–22)	132	1.83	0.79
25 (25–26)	109	1.97	0.86
30 (30–31)	94	2.10	0.93
35 (35–36)	83	2.20	1.04
40 (40–41)	72	2.40	1.08
43 (43–43)	63	2.51	1.15

### RF Homogeneity

**Figure 1:**
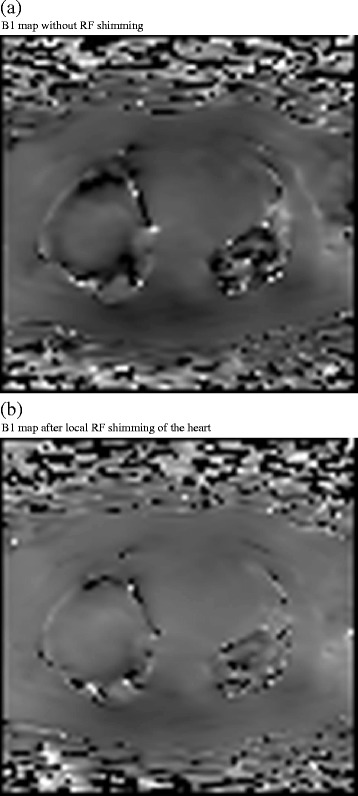
**Example of the effect of local RF shimming on B1 map in a volunteer.** Image **(a)** shows conventional RF transmission without RF shimming. Image **(b)** shows the use of dual source RF transmission with local RF shimming. The mean percentage of flip angle achieved was 74.6% with conventional RF transmission and 91.3% with dual source RF transmission demonstrating improved homogeneity.

Average B1 in the myocardium was higher for RF shimmed data. The mean percentage of the achieved flip angle in the myocardium was significantly higher and standard deviation lower at 91.1% ±1.9 with dual source RF transmission compared with 74.2% ±8.3 with single source mode (P = 0.011). Figure 1 shows B1 maps acquired in one volunteer (a) without local RF shimming and (b) with local RF shimming.

### Image quality and artefacts

**Figure 2:**
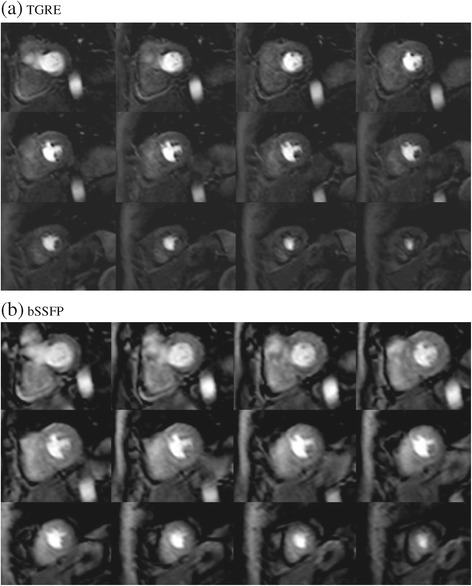
**Case example of a volunteer who underwent (a) TGRE 3D whole heart perfusion followed by (b) bSSFP 3D whole heart perfusion.** Both images had local RF shimming with dual source RF transmission applied and demonstrated similar image quality overall using *k-t* BLAST reconstruction. Image quality was scored as 3 in both sequences due to the presence of subendocardial dark band artefacts.

**Figure 3:**
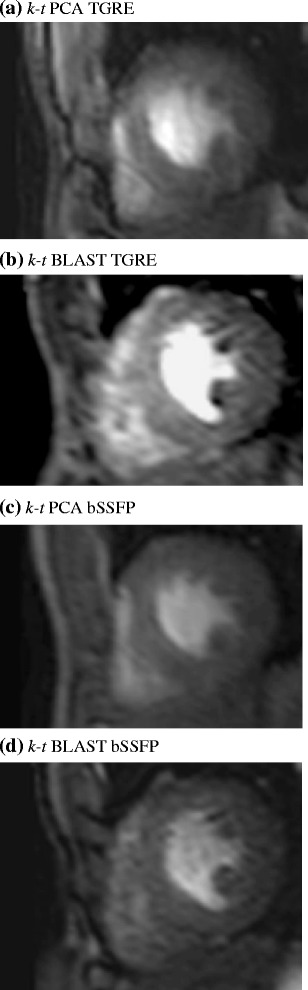
**Single slice Volunteer example of 3D spoiled gradient echo (TGRE) and balanced steady state free precession (bSSFP) acquisition.** Reduced subendocardial dark banding artefact was observed following reconstruction using *k-t* PCA **(a)** compared with standard *k-t* BLAST reconstruction **(b)**. Using a bSSFP sequence, SNR was demonstrated to be higher **(c)** and with *k-t* PCA reconstruction showed improved temporal fidelity compared with a standard *k-t* BLAST sequence with **(d)** similar picture quality.

Table 4 shows the image quality scores for the different sequences and reconstruction methods. The mean image quality (N = 15) was similar between the two methods at 3.0 for TGRE and 2.8 for bSSFP (p = 0.25) with *k-t* BLAST reconstruction and 3.2 for TGRE and 3.1 for bSSFP (p = 0.5) with *k-t* PCA reconstruction. Figure 2 shows an example with both pulse sequences. Breathing related artefact occurred in one data set with both sequences (6.7%), *k-t* related artefact (eg image flickering) once with both sequences (6.7%) and subendocardial dark rim artefact in two of the TGRE (13.3%) and four of the bSSFP (26.8%) data sets with *k-t* BLAST reconstruction. With *k-t* PCA reconstruction this was reduced to one TGRE (6.7%) and two bSSFP (13.3%) cases and image flickering was not seen with either of the sequences. There was more dark rim artefact with the bSSFP cases (2 volunteers and 2 patients vs 1 patient and 1 volunteer for TGRE). This improved after *k-t* PCA reconstruction (bSSFP - 1 volunteer, 1 patient, TGRE 0 volunteers, 1 patient) (Figure 3a-d).

**Table 4:** **Image quality scores for the** ***k-t*** **BLAST** ***and k-t*** **PCA images**

**Subject**	**bSSFP** ***k-t*** **BLAST**	**TGRE** ***k-t*** **BLAST**	**bSSFP** ***k-t*** **PCA**	**rTGRE** ***k-t*** **PCA**
1	3	3	3	4
2	2	3	3	3
3	3	3	3	3
4	2	3	3	3
5	3	3	3	3
6	3	3	3	3
7	3	3	3	3
8	3	3	3	3
9	3	3	4	4
10	3	3	3	3
11	3	3	3	3
12	3	3	3	3
13	3	3	4	4
14	2	3	2	3
15	3	3	3	3

### Signal to noise and signal homogeneity

**Figure 4:**
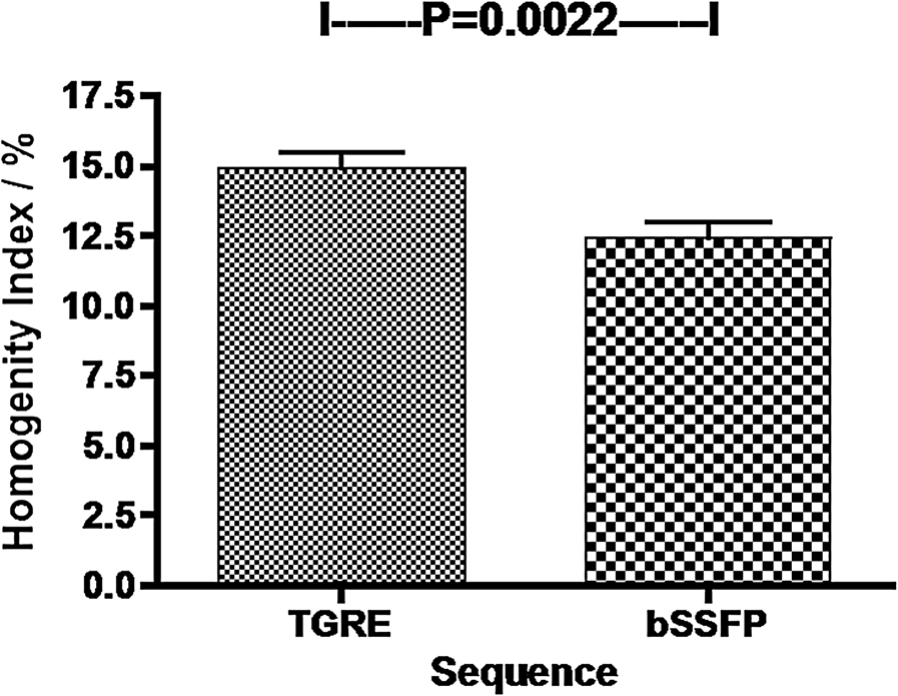
**Myocardial homogeneity index.** The lower index in bSSFP indicates significantly improved (p = 0.022) homogeneity of the myocardial signal compared with TGRE 3D whole heart perfusion.

**Figure 5:**
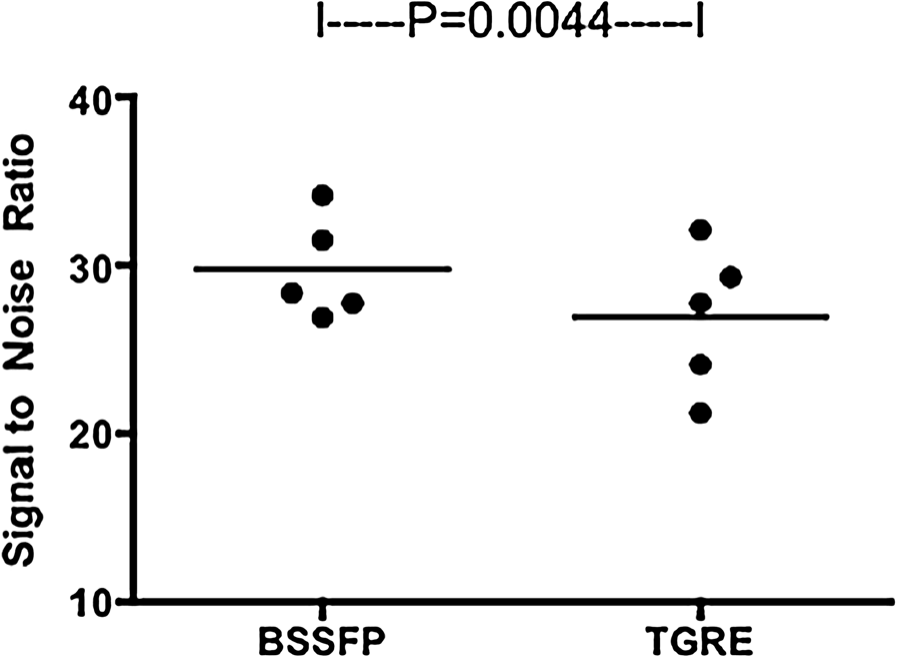
**Signal to noise ratio of bSSFP and TGRE 3D whole heart perfusion using** ***k-t*** **PCA reconstruction.**

The coefficient of variation of myocardial signal (“homogeneity index”) was significantly higher with TGRE than bSSFP (14.9%, versus 12.4%, p = 0.002), indicating more homogenous signal in bSSFP than in TGRE images (Figure 4). The mean SNR using bSSFP was 29.8 and with TGRE was 26.9 (Table 5 and Figure 5) (p = 0.045). The mean CNR using bSSFP was 23.2 vs 21.6 (P = 0.049) with TGRE.

**Table 5:** **Signal-to-noise and contrast-to-noise ratio in 5 volunteers**

**Volunteer number**	**Pulse sequence order**	**SNR**	**CNR**
**1.**	BSSFP	34.17	27.11
	TGRE	32.13	25.42
**2.**	BSSFP	31.53	27.12
	TGRE	29.33	26.29
**3.**	TGRE	27.78	21.44
	BSSFP	27.80	21.67
**4.**	TGRE	21.24	16.55
	BSSFP	26.92	18.30
**5.**	TGRE	24.10	18.20
	BSSFP	28.40	21.90
**Mean**	TGRE	26.9	21.6
	BSSFP	29.8	23.2
**P value**		0.045	0.049

### Stress acquisitions and patient studies

**Figure 6:**
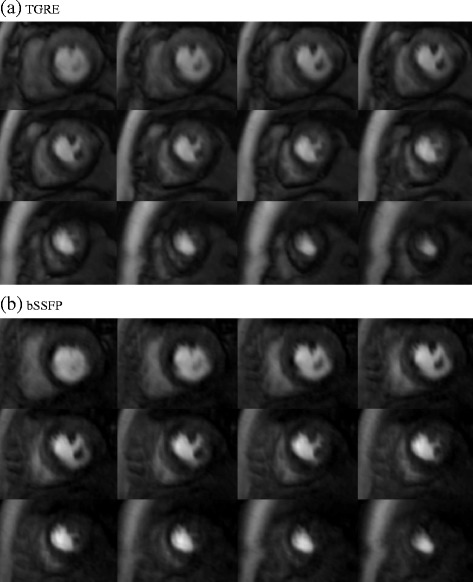
**Case example of a 79 year old patient who underwent (a) TGRE 3D whole heart perfusion followed by (b) bSSFP 3D whole heart perfusion.** Both images were reconstructed with *k-t* PCA and show significant ischemia of variable transmurality in the anterior and inferior wall extending from base towards the apex. Angiography showed an occluded proximal left anterior descending coronary artery (QCA = 100%) and a significant proximal stenosis of the right coronary artery (QCA = 85%). The circumflex artery was not considered flow limiting (QCA = 50%). Image quality was scored as 3 for both sequences using *k-t* PCA reconstruction.

Stress imaging was feasible and tolerated in all 5 volunteers and 5 patients without exceeding the maximum heart rate of the pulse sequences. Of the five patients, three had significant CAD on coronary angiography and all were correctly identified with both TGRE and bSSFP (Figure 6 – case example and Additional file 1 and Additional file 2).

## Discussion

This study has shown that the use of local RF shimming with dual source RF transmission significantly improves B1 field homogeneity and facilitates bSSFP 3D whole-heart myocardial perfusion imaging at 3T. Compared with a TGRE method, 3D bSSFP demonstrated significantly improved homogeneity, SNR and CNR but similar image quality. Artefact with bSSFP was more frequent but improved with *k-t* PCA reconstruction.

### High Field Strength

MRI at 3T has rapidly gained acceptance for many clinical applications [11,12]. B1 inhomogeneity and restrictions related to local energy deposition pose particular challenges for cardiac imaging at 3T [13]. Compared with other applications, myocardial perfusion CMR poses additional challenges at high field strength as susceptibility effects increase the B0 inhomogeneity and accentuate banding artefacts, requiring higher order shimming or other corrective methods [14].

Previous studies have reported the use of bSSFP for two-dimensional myocardial perfusion CMR at 1.5T [15-17]. bSSFP provides higher SNR, but at 3T has also been associated with increased off resonance artefact in perfusion CMR and a non-uniform flip angle distribution [8] causing higher local SAR (due to the higher excitation flip angle). At 3T, these issues are particularly relevant as SAR increases fourfold compared with 1.5T. A shorter TR is desirable for bSSFP but impacts gradient hardware and SAR constraint by increasing the RF duty cycle. Using longer RF pulses prolongs the TR, making off resonance artefacts more prominent.

In the present study compromises were made between the achievable flip angle and maximal heart rate of the acquisition. A flip angle of 35° represented the best compromise between shot duration and reasonable TR in both phantoms and volunteers.

### Dual Source RF Transmission at 3T using bSSFP

The dielectric effect is more pronounced at 3T than at 1.5T as the RF wavelength critically approaches the size of the body, influencing standing wave formation. As a consequence, flip angle variation at 3T is high and has previously been shown to range from 32-60% in the heart [18]. This effect contributes to reduced image homogeneity at 3T. Optimising RF shimming optimises power, amplitude, phase and waveform of each RF source to tailor the RF transmission to the patient’s anatomy. Use of two independent RF sources, as in this study, allows local RF shimming with consequent homogenisation of flip angle distribution and optimised SAR distribution within the myocardium, thus reducing local SAR peaks. The use of RF shimming has been successfully demonstrated in spine [19], abdomen [20] and breast [21]. In the heart, B1 calibration imaging with local RF shimming and dual-source RF transmission at 3T has been described for bSSFP cine and black blood imaging with improved image quality [8]. The technique was also shown to improve image quality and reduce the number of non-diagnostic segments for dobutamine stress CMR [22].

In the current study, dual RF transmission allowed shortening of the TR for bSSFP acquisition compared with single source RF transmission at the same flip angle. This permitted a relatively tight acquisition shot duration of around 200 ms for the bSSFP method, comparable to a previously described 3D TGRE myocardial perfusion method [4,5].

### SNR, CNR and homogeneity

Homogeneity can vary in part due to the distance of tissue from the surface [23]. Differences in signal intensity between the septum and lateral wall have previously been described in both bSSFP and TGRE imaging [6]. Although regional differences are thought to be mainly due to B1 inhomogeneities, improved image homogeneity with bSSFP compared with TGRE observed in this study may in part be attributed to the pulse sequence characteristics including dependencies of flip angle variation.

In this study, the use of bSSFP improved SNR, CNR and homogeneity of myocardial signal, compared with an equivalent TGRE method. The increase in SNR and CNR over TGRE was consistent with previous studies at 1.5T [6,16]. However, the magnitude in gain of SNR of bSSFP vs TGRE was not as marked as observed at 1.5T due to several factors including the differences in the coil design, relaxation times of the tissues, flip angles implemented, artefact, SAR limitations and field inhomogeneity [24].

### *k-t* PCA and *k-t* BLAST

Although detailed study was beyond the scope of this article, differences in *k-t* PCA and *k-t* BLAST were observed, consistent with previous reports [4]. In summary *k-t* PCA has a more efficient algorithm by defining spatial compartments within the 3D volume of a perfusion dataset. The number of overlapping signals is reduced and higher temporal frequency components are more accurately reconstructed. These effects are expected to improve temporal fidelity and reduce signal contamination artefact with *k-t* PCA and improve image quality. In this study, the use of *k-t* PCA reconstruction reduced the amount of artefact but this was not associated with a significant improvement of image quality.

### Image quality and diagnosis of CAD

Whilst increased signal of bSSFP acquisition should have advantages for diagnosis and analysis [6], in this study it did not translate into improved image quality. This was largely due to the higher rate of artefacts with bSSFP. The main artefact observed was subendocaridal dark rim artefact. Although in the 5 patients studied here, dark rim artefact did not affect the correct diagnosis of CAD, the presence of this artefact may lead to false positive reporting of ischemia.

### 3D Myocardial Perfusion CMR

3D acquisition has several potential advantages for myocardial perfusion CMR over the conventionally used 2D methods, including better coverage, higher signal-to-noise and fewer compromises regarding the cardiac phase in which data are acquired. Developing 3D myocardial perfusion CMR has been challenging and only recent technological developments including multi-element coils and spatio-temporal undersampling methods have permitted the very fast data acquisition required for 3D myocardial perfusion CMR. The use of bSSFP at 3T, as shown in this study, is a promising further development and warrants evaluation in larger patient populations. Another recent study has reported the use of bSSFP for 3D myocardial perfusion CMR, but with a different implementation and at 1.5T [25]. Giri et al. used no magnetization preparation but maintained the steady state by continuous application of the bSSFP kernel throughout the scan. This novel approach was feasible and demonstrated good signal characteristics. As the imaging was gated to mid-diastole, the acquisition was however limited to either single slice imaging or single slab 3D encoding and the read-out duration was longer than in our method, ranging between 300-380 ms under rest conditions. In our study it was the aim to keep the acquisition close to 200 ms comparable to previous published work, to avoid potential motion artefacts, which are more likely with a faster heart rate as seen during stress acquisitions.

### Implications for patient care

Three-dimensional myocardial perfusion CMR offers better myocardial coverage than conventionally used 2D methods. 3D bSSFP myocardial perfusion CMR at 3T potentially offers further improvement of signal characteristics and may enhance the use of three-dimensional myocardial perfusion CMR for clinical application.

## Limitations

The flip angle of 35 degrees we chose may not be optimal for bSSFP acquisition but was the maximal achievable within the restrictions of SAR, TR, heart rate and the desired acquisition shot duration. Higher flip angles may have improved the signal and image quality further and future studies should explore the optimal compromise between flip angle, image resolution and acquisition duration further. The differences in acquisition window may also account for some of the image quality discrepancies as it was anticipated that there would be more temporal blurring in the bSSFP group. The use of *k-t* PCA may have mitigated some of this effect however.

The maximal heart rate of 83 bpm is a limitation, although it was not exceeded by volunteers and patients in this study even during stress studies. In many patients, however, higher heart rates will be encountered. A reduction of the read-out duration or saturation pulse delay, or acquisition at every other heartbeat can be relatively easily employed to deal with higher heart rates but the impact on diagnostic accuracy will need to be evaluated.

Lastly, we recognise the reduction in TR associated with the use of dual source parallel RF transmission in this study is the consequence of many factors not least the particular SAR model used by the vendor of the MRI scanner used here. We would not consider this “optimal” as the resulting long acquisition time led to poor image quality. A number of approaches including dieletric cushions, post processing as well as a fixed anatomy dependent mode of RF shimming could have been used to improve B1 homogeneity. Many of these are vendor specific and were not available for the current study.

## Conclusion

Three-dimensional bSSFP myocardial perfusion CMR using local RF shimming with dual source parallel RF transmission at 3T is feasible and improves signal characteristics compared with TGRE. Image artefact remains an important limitation of bSSFP perfusion imaging at 3T.

## Additional files

Additional file 1:
**TGRE 3D Perfusion.**
Additional file 2:
**BSSFP 3D Perfusion.**
